# Gas Sensing by Bacterial H-NOX Proteins: An MD Study

**DOI:** 10.3390/molecules25122882

**Published:** 2020-06-23

**Authors:** Ahmed M. Rozza, Dóra K. Menyhárd, Julianna Oláh

**Affiliations:** 1 Department of Inorganic and Analytical Chemistry, Budapest University of Technology, Budapest Szent Gellért tér 4, H-1111 Budapest, Hungary; ahmed.rozza89@azhar.edu.eg; 2 Department of Biotechnology, Faculty of Agriculture, Al-Azhar University, Cairo 11651, Egypt; 3 Laboratory of Structural Chemistry and Biology & MTA-ELTE Protein Modelling Research Group, Institute of Chemistry, Eötvös Loránd University, Pázmány Péter sétány 1/A, 1117 Budapest, Hungary

**Keywords:** molecular dynamics, H-NOX, gas sensing, heme protein, sGC, diffusion, migration routes

## Abstract

Gas sensing is crucial for both prokaryotes and eukaryotes and is primarily performed by heme-based sensors, including H-NOX domains. These systems may provide a new, alternative mode for transporting gaseous molecules in higher organisms, but for the development of such systems, a detailed understanding of the ligand-binding properties is required. Here, we focused on ligand migration within the protein matrix: we performed molecular dynamics simulations on three bacterial (Ka, Ns and Cs) H-NOX proteins and studied the kinetics of CO, NO and O_2_ diffusion. We compared the response of the protein structure to the presence of ligands, diffusion rate constants, tunnel systems and storage pockets. We found that the rate constant for diffusion decreases in the O_2_ > NO > CO order in all proteins, and in the Ns > Ks > Cs order if single-gas is considered. Competition between gases seems to seriously influence the residential time of ligands spent in the distal pocket. The channel system is profoundly determined by the overall fold, but the sidechain pattern has a significant role in blocking certain channels by hydrophobic interactions between bulky groups, cation–π interactions or hydrogen bonding triads. The majority of storage pockets are determined by local sidechain composition, although certain functional cavities, such as the distal and proximal pockets are found in all systems. A major guideline for the design of gas transport systems is the need to chemically bind the gas molecule to the protein, possibly joining several proteins with several heme groups together.

## 1. Introduction

Gas sensing is an essential and crucial facet of cell signaling, regulating a vast array of cellular and physiological functions including respiration, cytoprotection, gas storage, neurotransmission, inflammation, gene transcription and cardiovascular homeostasis [1,2,3,4]. The binding of small diatomic gas molecules such as nitric oxide (NO), carbon monoxide (CO) and oxygen (O_2_) to intracellular sensing proteins triggers a conformational change that leads to versatile changes in the cellular metabolism [2,5].

NO is a key signaling molecule of human physiology which activates its receptor, soluble guanylate cyclase (sGC) [6]; impairment of the sGC-based signaling cascade has been linked to cardiovascular, neurodegenerative and inflammatory diseases. Nitroglycerol, a compound capable of releasing NO, has been used for more than a century for the treatment of angina pectoris, although its mode of action was not understood for long. This example highlights the therapeutic potential of small signaling molecules, but their clinical use has remained limited due to their gaseous nature, extensive reactivity, short half-life and systemic toxicity [7,8]. While small-molecule NO precursor compounds offer improvements over the direct delivery of NO, the practical application of these NO donor compounds remain hindered by their inability to target NO to a specific site [7]. Consequently, therapeutic use of gases like NO/CO/O_2_ requires specific delivery systems.

Selective binders can be used for trafficking gaseous ligands: H-NOX proteins (named Heme-Nitric oxide and OXygen binding domain) are one of the six major groups of heme sensor proteins whose bindings with the gaseous messengers cause changes in the downstream effector proteins, evoking various responses [9]. H-NOXs were already shown to be able to deliver O_2_ to hypoxic regions of solid tumors [10] and accumulate as a multimeric pattern in multiple intracranial glioblastoma models, thus improving the efficacy of radiation therapy [10,11].

However, in order to fully exploit the capacity of H-NOX proteins for delivering ligands, their selectivity and affinity need to be maximized. This requires a clear understanding of how ligand differentiation and affinity are governed by the protein matrix surrounding the heme. H-NOXs have a remarkably stable fold with moderate sequence identities (~30–35%), allowing for optimizing cavity structure and polarity for fine-tuning ligand preference—if the principles of such were clarified.

Coordination of gaseous ligands to the Fe (II)-protoporphyrin IX unit is governed by backbonding between the metal d and the ligand antibonding π* orbitals [12], resulting in an affinity ordering of NO > CO > O_2_ with K_D_ values of 1:~10^3^:~10^6^ commonly referred to as the sliding scale rule [13]. This affinity ordering, however, can eventually be modulated by the surrounding protein matrix. Further complication arises from the fact that these ligands bind to the heme group in a spin-forbidden process: in the course of the chemical binding step, the high-spin (quintet, with four unpaired electrons) ground state of the five-coordinate iron(II) system is converted to a low spin, six-coordinate system: doublet (one unpaired electron) in the case of NO and singlet (all electrons are paired) in the case of O_2_ and CO. The inversion of spins is formally forbidden and has a profound effect on the kinetics of binding [14,15].

Distal pocket size, shape and polarity are—unquestioningly—also distinct determining factors in ligand binding (in the case of heme proteins, the pocket containing the amino acid that directly anchors the heme to the protein matrix is called the proximal pocket, while the usually more spacious ligand-binding cavity at the opposing side of the heme is called the distal pocket). However, the polarity of the diffusing ligands is just the opposite of that of the bound ligands. Free NO has the largest dipole moment of the three, while O_2_ is completely apolar; but in contrast, iron-bound NO carries minimal charge on the oxygen, while O_2_ ligated to the heme is decidedly polarized. Thus, a distal pocket that is welcoming for the incoming gas molecule will necessarily be less apt to stabilize the ligand once it is coordinated to the heme iron. For example, the apolar pocket of facultative anaerobes like H-NOX from *Nostoc sp.* (*Ns*) or sGC is ideal for stabilizing O_2_ the gas molecule, but does not support the formation of stable Fe-O_2_ adducts. In contrast, the formation of a stable Fe-O_2_ bond can be observed in systems having a close reaching proton donor that is capable of stabilizing the partial negative charge appearing on the terminal oxygen once the Fe-O_2_ bond is formed. Examples include histidine in globins, or tyrosine in the case of obligate anaerobe H-NOXs from *Caldanaerobacter subterraneus (Cs)* (formally known as *Thermoanaerobacter tengcongensis* (*Tt))* or *Clostridium botulinum (Cb)* [16]. However, again, the distal pocket in itself cannot be the sole determinant of the ligand-binding process, since, for example, *Cb* and *Cs* H-NOXs, having the same residues reaching closest to the heme bound ligand, show characteristically differing affinities toward O_2_ with a K_D_(O_2_) of 5.3 × 10^−5^ M, K_D_(O_2_) = 4.4 × 10^−8^ M, respectively [17].

The proximal pocket, and especially the conformation and conformational freedom of the proximal residue as well as its basicity also have a direct effect on the reactivity of heme iron. In the case of sGC, for example, the proximal strain was shown to reduce affinity for O_2_ [18], which in itself might be a formidable contribution to the mechanism of O_2_ exclusion, and the NO-bound system is predicted to behave very differently from other related systems [19].

The tunnel system interlacing the entire protein matrix is the primary route of ligand entry to the binding site of H-NOXs and as such is of special importance in the design of ligand transport systems. However, controversial results have been obtained concerning the significance of the various diffusion routes. On the experimental side, xenon (Xe) saturation experiments in conjunction with X-Ray diffraction studies found bifurcated Y-shaped tunnels in *Ns* H-NOX, but no continuous pathway connecting the heme site and the solvent in *Cs* H-NOX showing that the fold of the protein is not the only determining factor of the ligand migration mechanism [20]. In contrast, based on the shape of the molecular surfaces, Wu et al. proposed that the major and most significant route for ligand entry to the heme group in all H-NOXs is the wide gateway in the plane of the heme group on the side of its propionates [9]; however, no such route was identified in the case of *Cs* H-NOX by molecular dynamics simulations [21]. These studies demonstrated that ligand access does not necessarily require additional channel systems to connect the heme site and the solvent, and that the different protein matrices of H-NOXs, in spite of the identical fold, possess different cavity structures, in which differing sidechain compositions lead to the formation of pockets of distinct positions, sizes and polarities, which could be important for their divergent functions.

**Figure 1:**
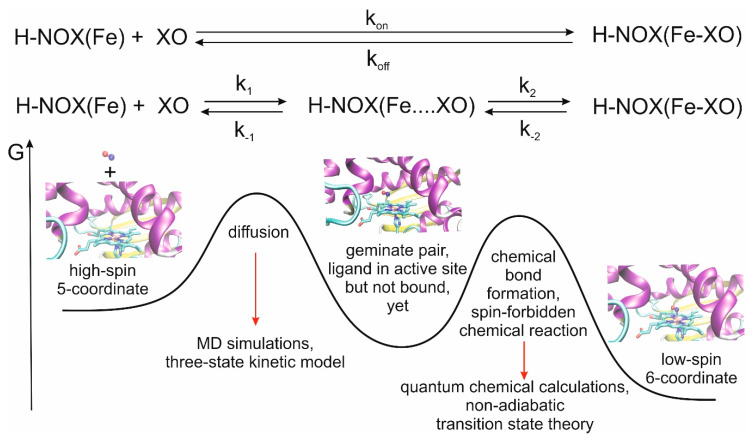
Ligand binding by Heme-Nitric oxide and OXygen (H-NOX) proteins and definition of the relevant rate constants. Experimentally, and usually, k_on_ and k_off_ are determined, while calculations allow us to study the steps individually and obtain the values of k_1_, k_−1_, k_2_ and k_−2_. The overall process can be divided into two major events: diffusion of the ligand to the active site and the formation of the bond between the ligand and the Fe(II) ion. Due to the nature of the two events, different methodologies are required for their proper description, and as the spins are inverted on iron in the course of the chemical reaction, a special form of transition state theory is needed for the derivation of the rate constant values [15,22].

We recently developed a multiscale model for the theoretical description of the CO and NO ligand-binding events of myoglobin, another histidine-ligated Fe(II)-heme protein and obtained very good agreement between calculated and measured rate constants [22]. A meaningful characterization of the overall process can be obtained by using a classical approach for the study of ligand diffusion and, in a separate step, quantum mechanical approach for bond formation between the ligand and the iron(II) center, which is a significant factor for the stabilization of the ligand in the distal pocket (Figure 1).

Here, we consider the first step of NO, CO and O_2_ binding of different bacterial H-NOXs and analyze in detail the diffusion kinetics, access and exit routes, and the cavities that may serve as traps or reservoirs for various ligands—to provide guidelines for the design of selective and effective transport machinery for diatomic gases, but do not consider the formation of the covalent bond between the ligand and the Fe ion. More specifically, we aim to answer the following questions: (1) How similar are the kinetics of the diffusion of the various gases in the studied proteins? (2) Is it sufficient to consider the direct route (opening at the heme propionates) for ligand entrance/exit OR should the tunnel system be also taken into account? (3) Are the channels specific to the fold (practically identical in all H-NOXs) OR matrix specific? (4) Are the ligand-binding pockets selective toward specific ligands, and if so, is this selectivity created by the local sidechain pattern or the global fold? (5) What kind of guidelines can be given for the design of NO/CO/O_2_-specific H-NOX transport systems in terms of pockets/cavities?

We chose three bacterial H-NOXs (*Ns*, *Ka* and *Cs*) for our study that differ in their ligand specificity and flexibility. *Ns* is an example of a facultative anaerobe, thus a selective NO binder. *Cs* is an obligate anaerobe that binds O_2_ with large affinity and qualifies as an O_2_-sensor [17], although it can also bind CO and NO. *Ka* is another example of facultative anaerobe microbes, however, it contains a unique glycine-rich hinge region, that plays a major role in flexibility and protein-protein interactions [23].

In order to answer the questions, we carried out 300–500 ns long molecular dynamics (MD) simulations on the three bacterial H-NOXs in solutions containing O_2_ or NO or CO or an NO-O_2_ mixture. We determined the rate constant for diffusion, analyzed the sensitivity of protein structure to ligand diffusion and identified diffusion routes and ligand storage pockets, and compared the various systems to get an overall picture of the major factors influencing the ligand transport properties.

## 2. Results and Discussion

First, we are going to describe our results obtained on the kinetics of the diffusion followed by the investigation of the tunnel systems and of the cavity structure of the studied proteins.

### 2.1. Structural Response of the Protein Structure to Gas Ligand Migration

**Figure 2:**
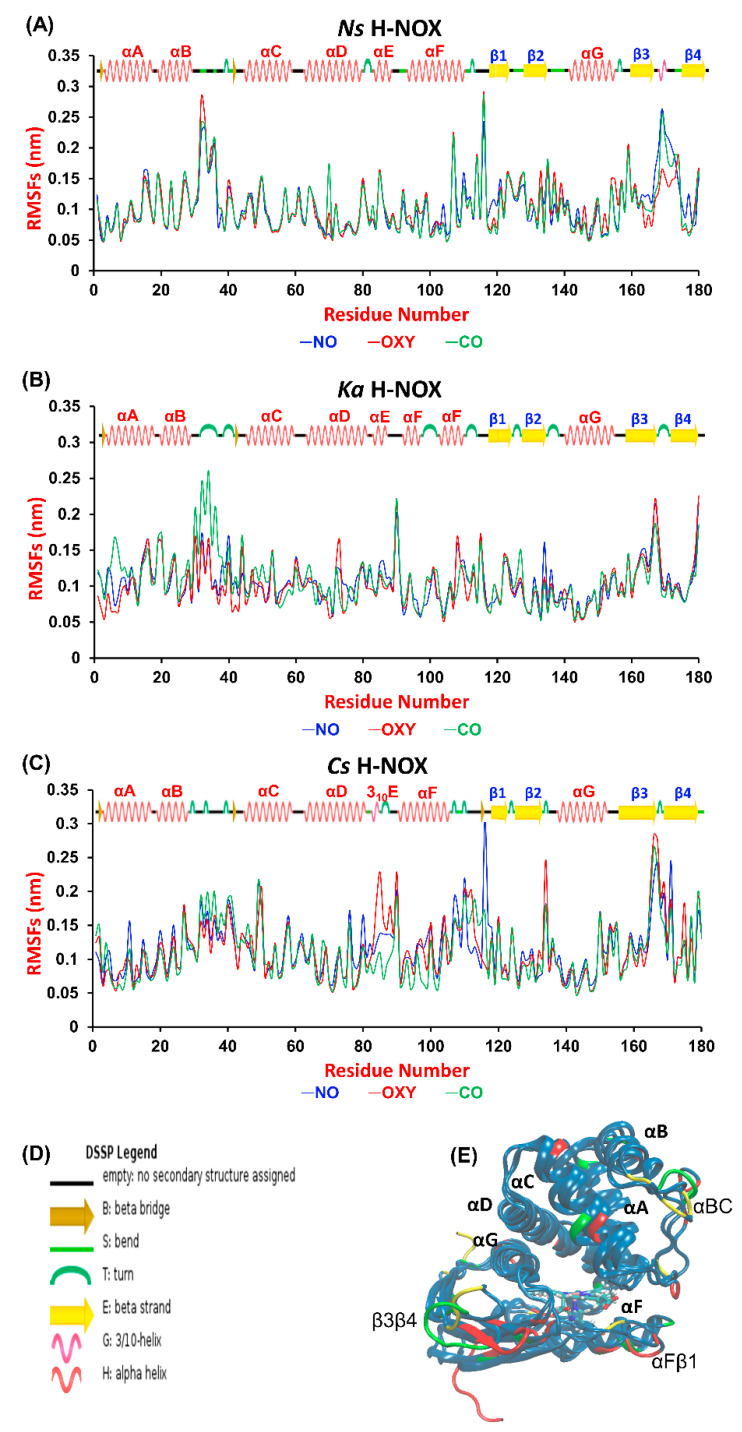
RMSF values computed per-residue from molecular dynamics (MD) simulations of gaseous molecule (NO, CO, O_2_) diffusion into (**A**) *Nostoc sp. (Ns)*, (**B**) *Kordia algicida* *(Ka)* and (**C**) *Caldanaerobacter subterraneus* (*Cs)* H-NOX. In the case of *Cs*, the RMSF values of the long tail (residues 181–188 that are not present in *Ns* and *Ka* H-NOX) are not shown to make comparison with other systems easier. (**D**) Labels used for the designation of secondary structure elements by DSPP in Figure 2A–C. (**E**) Superposition of the structure of the three H-NOX proteins. Regions with highest mobility have been highlighted by larger font size, and colored in green (*Ns*), yellow (*Ka*) and red (*Cs*).

A wide variety of experimental techniques (e.g., site-directed mutagenesis, EPR and Xe-binding X-ray diffraction) have been exploited to describe gas migration in the protein matrix [20,24,25,26]. Theoretical approaches, such as molecular dynamics (MD) simulations can provide complementary information on the dynamics of gas permeation through the protein matrix to obtain a full molecular-scale understanding of ligand transport [21,22,26,27]. First, we aimed at identifying those regions of the proteins that are most sensitive to gas ligand permeation. Therefore, we calculated the root mean square fluctuation (RMSF) per-residue averaged over time along the trajectories and plotted it against the residue number (Figure 2A, overall RMSD values are given in Table S1 in the Supplementary Materials). The most fluctuating regions (>0.15 nm) are identical for the three proteins: the αBC loop, the αFβ1 loop and the β3β4 loop, which connect the corresponding helices and β-sheets (see Figure 2C). These elements have been suggested to be catalytically significant, possibly regulating the entrance gate for gas ligands [21,28]. Our results do not show any systematic difference between the various systems, although we could identify sensitive regions in each protein to gas migration. In *Ns* ARG107 in αF seems to respond to the presence of all ligands, as its RMSF value exceeded 0.2 nm in all cases. Furthermore, the turn between β3 and β4 is very sensitive to the presence of CO and NO in *Ns*, but not to that of O_2._ The flexibility of the tiny 3_10_E helix, which is only found in *Cs*—the only oxygen sensor protein studied herein—increases considerably in the presence of oxygen. Out of the three proteins *Ka* H-NOX seems to exhibit the smallest flexibility, but its loop connecting αB and αC responds strongly to the presence of CO.

### 2.2. Diffusion Rate Constants for Gas Ligand Migration

After studying the response of the overall protein structure to the presence of gas ligands, we wanted to see whether any systematic difference exists between the rate constants for diffusion of the various gases in the three proteins. Due to the extended timescales needed for the modeling of the diffusion process, its realistic study only became possible since the appearance of GPU-accelerated MD simulations techniques. In recent years, reasonable rate constants for ligand diffusion to myoglobin [29], and to nitrogenase/hydrogenase enzymes [30,31,32] have been calculated.

**Figure 3:**
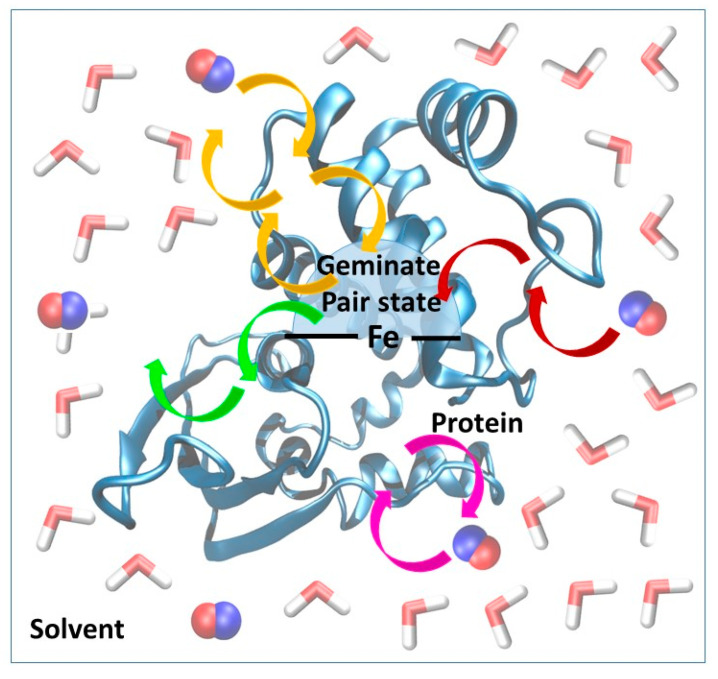
Schematic representation of the kinetic model used for the estimation of the rate constants for diffusion. The overall system has been divided into three parts: solvent phase (water molecules are shown in licorice), protein and geminate pair state (the geminate pair state is slightly smaller than the distal pocket). The heme group is represented by solid black lines. Ligand molecules (shown in van der Waals representation) may enter and leave the protein via various routes (indicated by yellow, green, pink and red arrows.). In the model, only those events are counted which represent the migration of the ligand from the solvent phase via the protein to the distal pocket and back to the solvent. Oscillations between the protein and the solvent/distal pocket are not taken into account.

We recently developed and validated against experimental results a three-state kinetic model for the study of ligand diffusion [22] that is much simpler than the complex Markov model used by Blumberger et al. [30,31,32]. The model groups the instantaneous position of the ligands into three different categories: in the solvent phase, protein matrix or distal pocket (Figure 3). During diffusion, ligand molecules enter the protein matrix from the solvent and gradually reach the distal pocket and could also return from the distal pocket to the solvent via the protein matrix. We call this a global in-and-out event. In equilibrium, the number of molecules reaching the active site from the solvent and returning to the solvent is equal per unit time. Thus, from the number of global in-and-out events and the length of the trajectory, the rate constants for diffusion into (k_1_) and out (k_−1_) of the protein can be derived. The equations used for the calculation and the definition of the various states (protein, distal pocket and solvent) are described in more detail in the method section. Naturally, the higher number of events observed, the more reliable the derived rate constants are. To this end, in the simulations, we used a relatively high gas concentration to ensure better sampling, as it was shown not to affect the rate constant values up to a concentration of about 0.5 M [26]. Furthermore, the reliability of the observed events can be checked by comparing trajectory sections: if the equilibrium has been reached one should observe twice as many events in 200 ns than in 100 ns, and the rate constants should be identical. This is what we did, and observed less than 15% variability in the rate of constant values.

Table 1 summarizes the calculated rate constants for diffusion as well as the experimental data for ligand binding. It is worth noting at this point that the experimental numbers can only be compared to the calculated results after careful consideration of the reaction mechanisms. In experiments, k_on_ and k_off_ are usually determined, which include both ligand migration and chemical bond formation. In contrast, here, we only address the ligand migration step (k_1_ and k_−1_, see also Figure 1). Depending on the relative values of k_1_, k_−1_, k_2_ and k_−2_, the bottleneck of the reactions can be determined. For example, in the case of myoglobin, we showed that diffusion is rate-limiting in the case of NO binding, while for CO binding both processes are partially rate-limiting and influence the overall value of k_on_ [22]. In contrast to binding, bond scission is the rate-limiting step of ligand release in all cases, as a consequence, no agreement between k_−1_ and k_off_ can be expected.

First, let us consider the reliability of our obtained rate constants. The case of NO binding provides an excellent opportunity for validation: it is diffusion-limited, as was suggested both by experiments and our own calculations on myoglobin. As a consequence, the overall rate constant is determined by the diffusion process, thus k_on_ = k_1_. Indeed, we find good agreement between calculated k_1_ and experimental k_on_ values: 6.5 × 10^8^ M^−1^ s^−1^ vs. 6.5 × 10^8^ M^−1^ s^−1^ for *Ns* and 3.8 × 10^7^ M^−1^ s^−1^ vs. 1.5 × 10^8^ M^1^ s^−1^ for *Cs*, respectively. In the case of CO, the second step is surely partially rate-limiting which makes a direct comparison impossible, while O_2_ only binds to *Cs*.

After having seen that the model is capable of yielding results in agreement with the experiment, we can compare the behavior of the various proteins and systems. A clear trend can be observed: the rate of ligand diffusion into the protein (k_1_) decreases in the O_2_ > NO > CO in all proteins, which most likely originates from the increasing size of the ligands and possibly increasing polarity. Contrarily, the k-_1_ values show no such trend arising from the fact that k_−1_ is influenced by the overall time that the ligand spends in the distal pocket, and this value varies a lot by protein and gas molecule. For example, the nonpolar distal pocket of *Ns* seems to stabilize O_2_ efficiently showed by the large occupancy ratio (in 91 ns out of the 300 ns long trajectory the pocket was occupied by O_2_), and as the polarity of the gas increases the time spent in the pocket decreases, in accordance with our expectations. The active site of *Ka* H-NOX prefers NO and that of *Cs* has an overwhelming preference for O_2_, again; this could be related to the O_2_-sensing character of *Cs* and the presence of a hydrogen-bond donor (TYR140) in the distal pocket.

If we analyze the behavior of a certain gas towards the various proteins, we see that the rates decrease in the *Ns* > *Ka* > myoglobin > *Cs* order. We note that the sampling of the in-and-out event is quite low for *Cs* which strongly suggests that migration into this protein is the slowest but also warns that the numbers should be treated with caution. The effect might be linked to the number of salt bridges found in the various systems. *Cs* is a heat resistant microbe and its H-NOX domain contains 20 salt bridges, significantly more than the five salt bridges found in H-NOXs of *Ns* and *Ka*. In addition, the smaller volume of the distal pocket of *Cs* compared to *Ns* [21] and *Ka*, and the presence of hydrogen bond triad found in *Cs* (H-bond of Trp9 and Asn74 to Tyr140 [16,21,33]) may contribute to the computationally predicted slower diffusion into *Cs*, which is in agreement with the slightly lower k_on_ value of NO in *Cs* than in *Ns*.

Until now, we have focused on the diffusion of a certain gas into a certain protein, but in reality, different sensor molecules might face a mixture of gases at the same time. We wondered if the presence of competitor molecules affects the diffusion properties of gases. This is an especially important question in the case of the human H-NOX protein, sGC, as it selectively binds NO even in the presence of multiple magnitudes of excess O_2_. Therefore, we carried out MD simulations in the presence of solutions containing both NO and O_2_. According to the best of our knowledge, this has not been done before. In this mixture, the concentration of the individual gases was half of that in the single-gas simulations, as we wanted to keep the overall gas concentration constant. Therefore, we expected half as many events per unit time compared to the one-gas simulations. We observed (see Table 1) that the value of k_1_ is not particularly sensitive to the presence of the other gas; in contrast, k_−1_ and the equilibrium constant K are strongly affected. This arises from the fact that the gases compete for the distal pocket and strongly influence each other’s residential time. Here, we can clearly observe that in the case of *Ns* H-NOX, if both O_2_ and NO are present, the preference for O_2_ decreases, compared to the single-gas simulations. Unfortunately, for *Cs*, we did not manage to observe any reasonable number of global in-and-out events that made the derivation of the rate constant values impossible. This probably arose as a consequence of several factors: (1) diffusion is slowest to *Cs* out of the three proteins; (2) we used a lower individual gas concentration than in the one-gas simulations; (3) as it will be shown later, in the case of *Cs*, the widest tunnel that could most easily channel various ligands at the same time is blocked, and therefore, gases, forced to choose from fewer possible inlets, especially hinder each other’s diffusion in this system.

### 2.3. Gas Migration Routes in H-NOX Proteins

**Figure 4:**
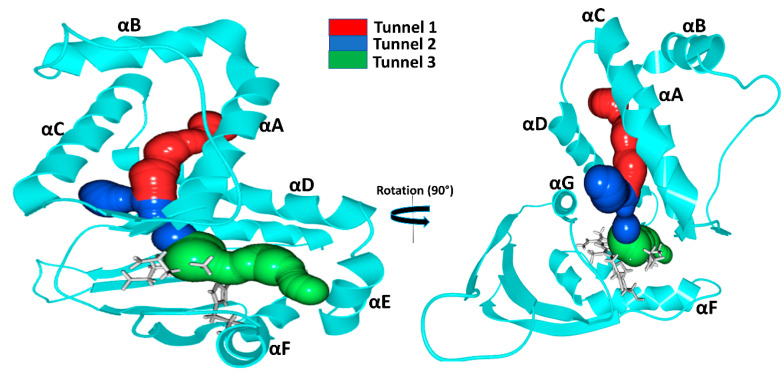
Identified gas migration tunnels. As the three-dimensional structures of the three proteins are very similar only the structure of *Ns* H-NOX is shown. The long, apolar tunnel is shown in red, while the shorter **Tunnels 2** and **3** are shown in blue and green, respectively. The heme group and the proximal histidine residue are shown in gray.

The tunnel system and protein dynamics together profoundly determine the routes taken by gas molecules from the solvent to the distal pocket. To get a clear picture, we visually inspected the inward/outward transport events that had previously been identified in our MD simulations and summarized in the previous chapter. We found three main diffusion routes that seem to be determined by the overall fold of the H-NOX proteins. Interestingly, each protein seems to use only two of the three possible routes and very rarely the third (Figure 4).

**Tunnel 1** extends between helices αA and αD from the solvent to the distal heme pocket. It is primarily composed of the sidechains of hydrophobic amino acids (residue numbers for *Ns*: VAL5, ILE9, MET12, ILE13, THR48, VAL52, LEU66, LEU67, PHE70 and LEU141) and has been previously suggested by Xe-binding experiments [20]. **Tunnel 2** is the shortest route to the heme lined by helices αC, αD and αG and tunnels the gas ligand directly from the external solvent environment towards the heme propionic moiety and then to heme-FE. It is primarily bordered by polar residues (residues numbers for *Ns*: MET1, THR48, TYR49, LEU67, ARG138, CYS139, GLY140 and LEU141). These routes intersect at LEU67 position forming a bifurcated Y-shape channel leading to the heme-binding cavity [21]. **Tunnel 3** is bordered by three helices: αA, αD and αF (*Ka*: ILE4. VAL77, ILE78, SER81, TYR82, VAL107 and TYR111).

In the case of *Ns* H-NOX, we observed, in accordance with previous results, ligand migration primarily through **Tunnels 1** and **2** (Figure 4). **Tunnel 3** is blocked by the bulky residue TRP74 bordering the distal pocket: the indole ring of TRP74 is perpendicular to the heme plane, creating enough steric hindrance to hamper gas access. Additional hindrance arises from the hydrogen bond between TRP74 and heme group (see Figure S1 in the Supplementary Materials). In silico mutation of TRP74 to ASN was shown to open this tunnel for ligand migration as well [21].

*Ka* H-NOX is the least studied among crystallized H-NOXs. Our results show that ligands primarily use **Tunnels 1** and **3** for migration. Similarly to *Ns,* it has an apolar elongated access tunnel (**Tunnel 1**: ILE5, PHE9, LEU12, VAL13, PHE17, MET48, LEU52, LEU66, LEU67, THR69, TYR70, MET139) and similarly to *Cs* a slightly polar short route lined by αA, αD and αF (ILE4. VAL77, ILE78, SER81, TYR82, VAL107 and TYR111 see Figure 4) leading to one side of the heme group. For this system, the direct **Tunnel 2**, previously suggested to be universal for all H-NOXs, was not observed [9]. Closer inspection showed that this tunnel is blocked at both ends in *Ka*. At the entrance from the solvent phase, PHE45 establishes a cation–π with the vertically aligned ARG137, and the exit of the tunnel at the distal pocket is blocked by two interacting methionine residues (MET 1and139) (see Figure S2 in the Supplementary Materials).

In *Cs* H-NOX ligands primarily move through **Tunnels 2** and **3** (Figure 4), but not the longer, apolar tunnel (**Tunnel 1**). Both of these routes are short and provide easy access to both the distal and the proximal sides of the heme. These tunnels may play a major role in the reactivity of sGC [36], *Shewanella oneidensis* (*So*) H-NOX [9] and *Vibrio cholera* (*Vc*) H-NOX for which ligand-binding to the proximal side has also been suggested. **Tunnel 1**, the long apolar tunnel has been visited by ligands at its entrance towards the solvent phase; however, the exit to the distal pocket remained fully blocked by a hydrogen bonding triad (TYR140 with sidechains of TRP9 and ASN74). Earlier in silico efforts to open this tunnel by double mutation (TYR140PHE and ASN74LEU) of the residues failed. When we further examined this tunnel, we found that there are two bulky residues along this tunnel: TRP9 and TRP67 forming extensive hydrophobic interactions, and we speculate that their presence also significantly contributes to the blocking of the tunnel beside the hydrogen-bonded triad (see Figure S3 in Supplementary Materials).

Comparison of *Ka* with *Cs* and *Ns* H-NOXs systems clearly shows that the overall fold primarily determines the tunnels, but the specific protein matrix has a significant effect on their accessibility, through hydrogen bonding networks, steric hindrance, cation–π interactions or a combination of these. Potentially salt bridges may block tunnels as well, but we could not find any direct proof for this in our systems.

Our results also contradict the suggestion that the major place of ligand entry is the entrance at the propionic groups of the heme group in all H-NOXs. We clearly see that it is not the length of the tunnel that primarily determines the rate constant for diffusion, much more so the spaciousness of the tunnel and the presence of spacious pockets along the route which provide opportunities for ligands to pass next to each other similarly to railroads with one track.

### 2.4. Pockets and Cavities Identified in H-NOX Proteins

There are two major types of protein concavities: pockets (in principle found on the surface, but the distal pocket is buried) and cavities (usually buried). However, considerable controversy is found in the literature regarding the naming; therefore, we will call all concavities “pockets”. Pockets play a significant role in biological functions such as ligand-binding and trapping. The active site (or in the case of heme proteins the distal pocket) is usually in the center of interest; however, other cavities/pockets may play a role in gas storage. Therefore, we first obtained global descriptors of the interactions between gas molecules and the protein matrix (see Table 2), then the properties of individual pockets have been analyzed (selected properties are given in Table 3, while an extensive comparison of the pockets for all systems are given in the Supplementary Materials in Tables S2–S4).

The average number of gas molecules found in the protein matrix shows distinct differences between proteins: the capacity to store gas molecules decreases in the *Ns > Ka > Cs* order, and this is most pronounced for NO. It is in accordance with the native function of the gas sensors: *Ks* and *Ns* are NO-sensors that do not coordinate oxygen. For these two proteins the amount of gas stored in the protein decreases in the NO > O_2_ > CO order, but interestingly, and possibly related to its O_2_-sensor function, *Cs* H-NOX has a preference for O_2_. Similar conclusions can be deduced from the average number of protein contacts summed over all gas molecules. A higher number of protein contact/gas molecules implies that gas molecules are trapped in pockets more buried within the matrix, which is highest in the case of *Cs*. Moreover, we found that the ratio between the number of protein contacts to a single-gas molecule was higher for *Cs* H-NOX than that of *Ka* and *Ns* H-NOXs which suggest the presence of less spacious pockets and possibly higher packing density.

We quantitatively analyzed the accessible-binding spots positioned through the three gas migration routes (Table 3). Altogether eight pockets have been identified for *Ns* H-NOX, six for *Ka* and seven for *Cs* H-NOX. For the trajectories of NO-diffusion, we have added the maximum and the average number of gas molecules in the pockets as well. It is obvious that *Ns* and *Ka* H-NOX have several spacious pockets that can accommodate several ligands at the same time, while *Cs* has only smaller pockets. This is in line with our previous proposition that wide spots facilitating ligand exchange in the two directions are needed for faster diffusion. It is also worth estimating the average number of gas molecules found in the pockets (thus summing the numbers in Table 3) and comparing them with the average number of gas molecules in the various proteins in Table 2. The comparison of NO molecules shows a larger difference for *Ns:* ~10 vs. 17.6 and for *Ka:* ~9 vs. 16.4, but a reasonable agreement for *Cs:* 7.2 vs. 9.4. The question arises where the molecules could be found that are not in the identified pockets. We believe that molecules temporarily can access other parts of the protein but do not reside there long enough to call those parts pockets (e.g., the route towards the buried pocket in *Ka*) or they can be temporarily associated with the surface of the proteins, but quickly changing position.

Let us turn our attention towards the identified pockets. Along **Tunnel 1** (lined by αA and αD) in *Ns* and *Ka*, two hydrophobic binding spots have been identified that are not only shared by all three gas molecules of this study, although with differing occupancies, but were confirmed as binding sites by Xe trapping experiments (Xe2 and Xe3) in the case of *Ns* H-NOX (PBD id: 3TFA and 3TF9). A polar entrance spot is associated to **Tunnel 2** (lined by αC, αD and αG) in *Ns* and *Cs*, which directly connects to both the proximal and distal pockets, and was also confirmed experimentally (in the case of *Cs* H-NOX (PBD id: 3TF1, “Xe2”)). **Tunnel 3**, similarly to **Tunnel 2** is very short and it is lined by αA, αD and αF. It has been frequently taken ligands both in *Ka* and *Cs* H-NOX as shown by the data in Table 3.

*Ka* and *Cs* possess a buried spacious hydrophobic pocket situated between the distal and proximal lobes near the proximal pocket (formed by αF, αG and the proximal β-sheet). This spacious pocket (BP1) in *Cs* is most frequently occupied by O_2_ (69.5% of snapshots there is at least one oxygen molecule inside); neither NO, nor CO binds to this pocket. While in the *Ka* system, BP1 was most selective toward the NO ligand (94.8% of the snapshots). Two small hydrophobic pockets (BP2 and P1SP) in *Cs* H-NOX were demonstrated to be CO-specific with 99.3% and 84.4% of snapshots. BP2 is near the distal, while P1SP is near the proximal pocket. Another buried hydrophobic pocket has been recognized in *Cs* H-NOX, which is mostly sampled by the NO ligand. Interestingly, this pocket contained one of the two Xe molecules trapped in *Cs* H-NOX under 6 atm of pressure (PBD id: 3TF1, “Xe1”) [20,37].

Apart from the accessible-binding sites that are located along the diffusion routes, only the *Ns* H-NOX system displayed three surface pockets occupied by various gas ligands. These pockets are more polar than buried ones thus water molecules can also access them. Two (P1SP and P2SP) are in the proximal side, and another one is positioned at the distal side (DSP) lined by αBC loop, αB and αC. P2SP appeared to be NO-specific, trapping an NO molecule in 99.3% of trajectory frames.

In the Supplementary Materials, in Tables S2–S4, we give detailed information on several parameters of ligand diffusion and binding. It is obvious that there is an inverse relationship between the time spent in the pocket and the number of in-and-out events. In most cases, ligands spend little time in pockets which can be easily accessed, and spend more time in pockets with hindered access. On average, the residential time is in the range of 20–200 ps, but in the case of the proximal pocket of *Ka*, much longer time has been found: in the range of 2–8 ns for CO and NO. One might expect that buried pockets are better places for storing ligands in terms of residential time, but interestingly, this is not fully proven. In the case of the distal surface pocket of *Ns* H-NOX, the residential time is 2–4 ns for CO and NO. This pocket is bordered by amino acid residues of various characters. Hydrophobic and/or bulky groups such as PHE36 and PHE37 are included as well as amino acids with positively or negatively charged sidechains such as LYS26 and ASP35. This suggests that composite pockets are better for storing ligands, which is also supported by the fact that the residential time of ligands in the non-polar cavities along **Tunnel 1** is significantly shorter both in *Ns* and in *Ka* H-NOXs (on average shorter than 150 ps).

### 2.5. Guidelines for Designing Gas-Transporting Proteins

We asked in the introduction whether guidelines could be given for the design of protein systems in order to transport gas molecules and whether such systems could be designed. The results discussed until now show that all of the studied H-NOX proteins are capable of storing a reasonable number of gas molecules at the same time. It is also apparent that the residential time of ligands in these spots varies greatly: from ~10 ps to 8 ns and that composite pockets including both charged residues and bulky groups are most suited for storing ligands for longer periods of time, thus this property is designable. However, would this be enough for the efficient transport of gases?

In order to answer such a question, let us consider the gas release by hemoglobin, which is the most prominent gas-transporting protein in the human body. The rate constant for the release of oxygen (k_off_) of hemoglobin 1 is 61.1 s^−1^ [38]. Let us make a thought experiment and suppose that the rate constant is due to ligand release only from the pocket and no bond breaking takes place. How long residential time in a pocket would result in a rate constant of 61.1 s^−1^? In order to answer this question, we could use Equation (5) of the method section and use the data given for *Ns* H-NOX in the Supplementary Materials. By considering the number of events to be one, and substituting Avogadro’s number, protein concentration (0.044 M), cell volume (3.78 × 10^−22^ dm^3^), we get 1.64 ms for the residential time, which is orders of magnitude bigger than the longest time we observed (around 8 ns).

Therefore, the major guideline for designing transport proteins is to increase the residence time of ligands in the pockets into the region of at least ms. This might be achieved by the selective design of the pockets, but the covalent linking of the ligand to the heme group could also be considered and creating e.g., fused proteins with several heme groups could increase the transport capacity, as was exploited in a recent patent [39].

## 3. Materials and Methods

### 3.1. Molecular Dynamics Simulation

The crystal structures of *Ns*, *Cs* and *Ka* H-NOX domains were obtained from the protein data bank under acquisition numbers 2009, 1U55 and 6BDD, respectively. The protonation state of the titratable residues under neutral pH was predicted using the H++ web server [40,41,42]. Protonation states of HIS residues were determined after visual inspection of the structures: in *Ns* and in *Cs* all HIS residues were protonated on δN, and in *Ka* HIS 104, HIS141 and HIS 153 were protonated on δN and HIS102 and HIS140 were protonated on εN. The protein was solvated by OPC [43] water molecules arranged in a dodecahedral box, with the edge of the box being at least 12 Å away from any point of the protein. Total charge was neutralized using Na^+^ and Cl^−^ ions. The structure of the prepared system was energy-minimized in order to eliminate bad initial contacts. MD simulations were carried out as implemented in GROMACS [44] using the CHARMM27 force field for the protein atoms, and recent three-site parameter models for the NO [45], CO [32] and O_2_ [30] molecules (parameters are also given in the SI). A Combination of these parameters sets for the diatomic ligands and the CHARMM27 force field was previously successfully used for the study of diffusion as evidenced by the results in Refs, [30,32,45]. Energy minimization of starting structures was followed by sequential relaxation of constraints on protein atoms in three steps and an additional NVT step (100 ps) to stabilize pressure. Trajectories of NPT simulations at 310 K and 1 bar were recorded for further analysis (collecting snapshots at every 4 ps).

The final structures obtained from the equilibration of gas-free models (the midstructure of the most populated cluster (derived based on the conformation of the main-chain)) were used as the starting conformation for further simulations. Forty NO or CO or O_2_ molecules (or 20–20 NO and O_2_ molecules in the case of mixtures) were placed in the bulk phase by first creating solvent holes and then inserting the gas molecule into these, resulting in an gas concentration of approximately 0.13–0.17 M (see Table S5 for more details). This concentration is high compared to the physiological concentration of NO or CO, but using this higher concentration, a better sampling of diffusion events could be achieved. Furthermore, similarly high concentrations were used by Blumberger et al. showing that up to about 0.500 M the kinetics of ligand diffusion is independent of the concentration [26,29]. The overall architecture of the protein did not change in the simulations, as it is also witnessed by the calculated backbone RMSD values that are reported in the SI in Table S1 (all values are below 0.165 nm for *Ns* and *Ka* and below 0.29 nm for *Cs* due to the presence of a long, almost freely moving tail).

### 3.2. Diffusion Rate Calculation 

In order to obtain the rate constants for diffusion we used a three-state model. We assigned one of the following three states to each NO/CO/O_2_ molecule in every frame of the productive MD simulations. We measured the distance ($d\left(r_{\mathrm{XO},i}^{\mathrm{mc}}\;{-\;r}_{\mathrm{Fe}}\right)$) between the heme Fe (r_Fe_) and the center of mass ($r_{\mathrm{XO},i}^{\mathrm{mc}}$), of any gas molecule and if it was less than 3.5 Å, the ligand was said to be close enough to form a geminate pair with the heme Fe (please note that the volume of this “geminate pair” state is smaller than the actual distal pocket). The XO molecule was assigned to be in the “protein” if it was not forming a geminate pair with Fe, but it was within 6 Å from any of the protein heavy atoms ($r_{\mathrm{protein},j}$). In all other cases, the ligand was assigned to be in the solvent phase. The definitions for the three states:

(1) $$“\mathrm{geminate}\;\mathrm{pair}”:\;d\left(r_{\mathrm{XO},i}^{\mathrm{mc}}{-r}_{\mathrm{Fe}}\right)\;<\;3.5\;Å$$

(2) $$“\mathrm{protein}”:\;d\left(r_{\mathrm{XO},i}^{\mathrm{mc}}{-r}_{\mathrm{Fe}}\right)\;\geq \;3.5\;Å\;\mathrm{and}\;d\left(r_{\mathrm{XO},i}^{\mathrm{mc}}{-r}_{\mathrm{protein},j}\right)\;<\;6\;Å$$

(3) $$“\mathrm{protein}”:\;d\left(r_{\mathrm{XO},i}^{\mathrm{mc}}{-r}_{\mathrm{Fe}}\right)\;\geq \;3.5\;Å\;\mathrm{and}\;d\left(r_{\mathrm{XO},i}^{\mathrm{mc}}{-r}_{\mathrm{protein},j}\right)\;\geq \;6\;Å$$

Using these, the number of the events when an XO molecule entered from the solvent phase to the distal pocket (global *“in”*-event: solvent→protein→geminate pair state) could be determined, as well as the number of events when the XO molecule left (global “out”-event: geminate pair state→protein→solvent).

In equilibrium the number of global in-and-out events are equal, and the rate constants for diffusion can be derived with the aid of Equations (4) and (5):

(4) $$\frac{d{\left[\mathrm{XO}\right]}_{\mathrm{in}}}{\mathrm{dt}}=-k_{1}[\mathrm{Protein}][\mathrm{XO}]\;=\frac{{№i}_{\mathrm{in}-\mathrm{out}}}{N_{A}}\cdot \frac{1}{t\cdot V}$$

(5) $$\frac{d{\left[\mathrm{XO}\right]}_{\mathrm{out}}}{\mathrm{dt}}=-k_{-1}[\mathrm{Protein}\cdots \mathrm{XO}]\;=\frac{{№i}_{\mathrm{in}-\mathrm{out}}}{N_{A}}\cdot \frac{1}{t_{\mathrm{XO},\mathrm{pocket}}\cdot V}$$

where №_in-and-out_ refers to the global number of events when an XO molecule reaches the active site from the solvent phase and returns there, N_A_ is Avogadro’s number, *t* the simulation time and t_XO,pocket_ the time that the ligands spend in the distal pocket, V the average volume of the unit cell (~3.7 × 10^−22^–5.0 × 10^−22^ dm^3^, see Table S5) during the simulation, respectively. After substituting the corresponding values into Equations (4) and (5), one can obtain the values of k_1_ and k_−1_.

### 3.3. Identification of Tunnels and Cavities 

Over the last 300 ns, as an equilibrated part of the MD trajectory, we defined the heavy atoms that are in contact with gas molecules at a distance cutoff (4.0 Å) between protein-heavy atoms and the dummy atom of the gas molecule. Protein-heavy atoms were additionally ranked in descending order according to the number of contacts. The upper 50% of all atoms identified as making contacts with gas molecules were visualized and spatially close-lying residues were identified as forming pockets. Finally, using a Gromacs script, the occupancy of pockets by the gas molecules was determined with a cutoff distance of 3.5 Å between the centroid of the pocket and the dummy atom of the gas molecule, and the average time spent in the pocket, the number of in-and-out events were quantitatively computed.

## 4. Conclusions

Gas sensing is a complex process that involves the migration of the gaseous ligand to the active site of the protein from the environment, the chemical bond formation and finally also ligand release. As gas sensing H-NOX proteins were suggested as suitable candidates for transporting gaseous molecules, we set out to investigate their dynamic response to the presence of gas molecules, focusing on the migration events of gaseous ligands into and out of the H-NOX protein matrices.

We compared three systems with decidedly different ligand affinities (and flexibility) in a comprehensive analysis and we concluded that the basic fold is quite similar and the flexible regions are also in the same segments in the three systems despite the low homology of their sequences. We could identify three conserved tunnels in all three proteins, the accessibility of which, however, differ in the three. Neither protein relies on a single short access route connecting the heme and the solvent, as was suggested earlier [21], but utilizes at least two of the three tunnels.

We also showed—for the first time—that the rate of ligand migration follows the same ordering (O_2_ > NO > CO) in the three systems. Diffusion into the protein, characterized by k_1_ is much less sensitive to the specific protein matrix than ligand egress (k_−1_), the differences created in both the size and polarity of the cavities by the different sidechain composition of the three proteins significantly influence the kinetics of the diffusion out of the protein. This is nicely demonstrated by the oxygen ligand spending the longest time within the protein matrix of the oxygen sensing *Cs* protein while *Ns* and *Ka*, which sense mainly NO, are more effective in retaining both CO and NO than oxygen.

By modeling the gas-binding event using a gas mixture we were the first to show that they influence each other’s trapping in the distal pocket.

We have also shown that the overall rate of gas diffusion is influenced by the total number of intramolecular H-bond/salt bridges of an H-NOX protein: there are many more salt bridges in *Cs* H-NOX than in the other two systems and this has a remarkable effect on ligand migration. Actually, this is a very simple parameter, but has a profound effect and should be considered.

By searching for reasons for the selectivity differences between the oxygen sensing *Cs* H-NOX and NO-sensing *Ka* and *Ns* H-NOXs we found that in the case of *Cs* H-NOX the whole protein matrix seems to accommodate oxygen much better than the other ligands (as shown in Table 2). *Cs* H-NOX is the only protein matrix that slightly prefers oxygen over NO (or CO), while the other proteins show a very strong preference for NO. We found several factors that probably contribute to creating this difference. *Cs* H-NOX is the most tightly woven protein matrix of the three, rigidified by a great number of intramolecular interactions, which creates narrower, less pliable channels and thus a preference for the smallest ligand: oxygen. Additionally, in *Cs* H-NOX, the spacious, apolar **Tunnel** 1 which would provide an ideal escape route for the apolar oxygen ligand is blocked, so O_2_ is forced to enter and exit through the decidedly more polar side tunnels. Oxygen is fast to reach the distal binding spot in all three proteins, but in the case of *Ns* H-NOX and *Ka* H-NOX, it also leaves the distal pocket rapidly. In the case of *Cs* H-NOX, its detainment provides enough time for oxygen—the weakest binder of the three studied gases—to chemically bind to the heme group.

Our results suggest that several properties of H-NOX proteins could be tuned when the design of gas transport proteins is considered. The highly conserved global fold creates rather similar tunnel systems in H-NOX proteins, but the accessibility of these is determined by the individual sequences—thus it is a designable property. We identified several ligand-selective pockets in the various protein matrices, demonstrating that the local environment can be tailored to enhance selectivity. We also showed that an intricate sidechain-based H-bond system could greatly hinder the movement of ligand molecules within the protein matrix. Our results indicate, that since the fold of H-NOX proteins is remarkably stable, it should be possible, through selecting or blocking channels and redesigning the nature of surface-close polar and buried apolar pockets, to create selective and effective gas transport vehicles based on the proteins of the H-NOX family, and the design of systems including various heme groups is advised for longer storage times.

## Figures and Tables

**Figure 5:**
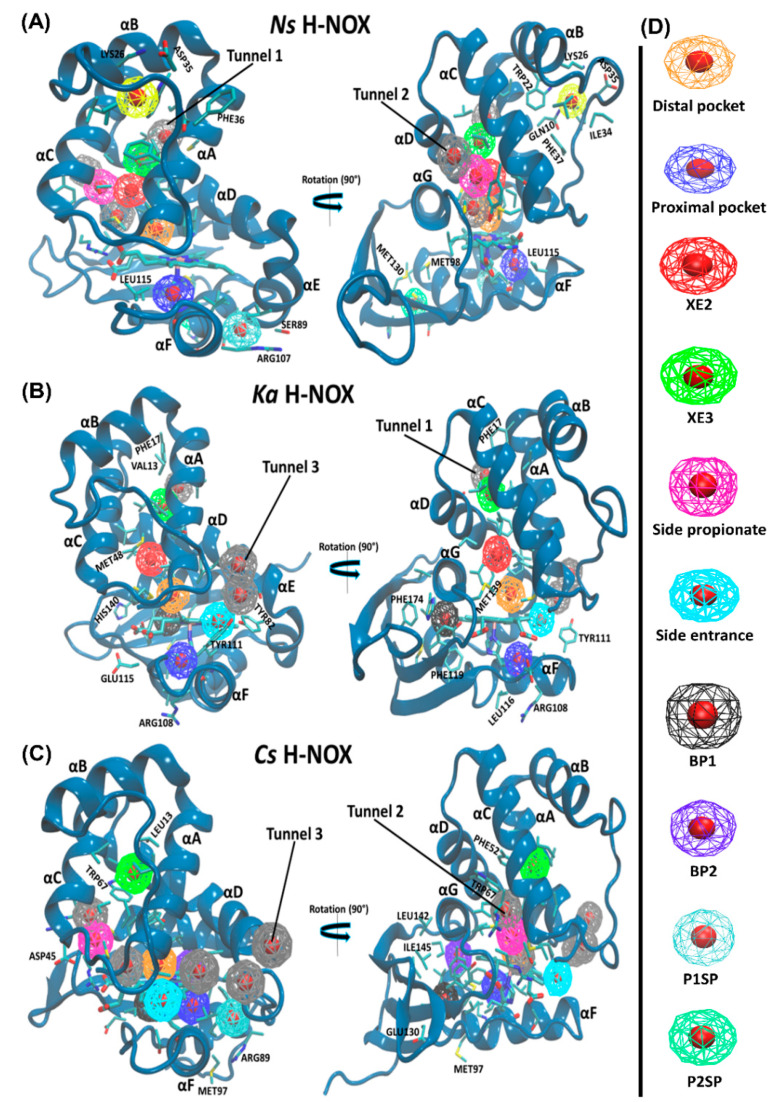
Gas-binding cavities in the studied H-NOX systems. Color coding used for the cavities are shown in the panel on the right.

**Table 1:** Calculated and experimental rate constants and equilibrium constant for the diffusion of diatomic gases into and from the active site of studied H-NOXs. Number of global in-and-out events ($№i_{in-out})$. The trajectory length used for the simulation and the time that the ligands spent in the distal pocket are given in ns. For comparison, previous results for myoglobin are also added.

Ligand	$№i_{in-out}s$	Simulation Time (ns)	Ligand in “Geminate Pair” State [ns]	k_1_ (M^−1^ s^−1^)	k_−1_ (s^−1^)	K(k_1_/k_−1_) (M^−1^)	Exp. k_on_ (M^−1^ s^−1^)	Exp. k_off_ (s^−1^)
*Ns* H-NOX								
O_2_	83	300	91	1.6 × 10^9^	9.1 × 10^8^	1.8	N/A	N/A
NO	34	300	55.8	6.5 × 10^8^	6.0 × 10^8^	1.1	3 × 10^8^ [9]	5 × 10^−2^
CO	14	300	16.4	2.7 × 10^8^	8.5 × 10^8^	0.3	3 × 10^6^ [9]	3.6
**NO/O_2_ mixture**								
O_2_	23	200	15.17	1.3 × 10^9^	1.5 × 10^9^	0.86		
NO	9	200	12.1	5.1 × 10^8^	7.5 × 10^8^	0.68		
*Ka* H-NOX								
O_2_	57	300	21.3	1.1 × 10^9^	2.7 × 10^9^	0.4	N/A	N/A
NO	33	300	60.2	6.4 × 10^8^	5.5 × 10^8^	1.2	N/A	N/A
CO	7	300	0.27	1.4 × 10^8^	2.6 × 10^10^	0.0052	N/A	N/A
**NO/O_2_ mixture**								
O_2_	37	200	19	2.1 × 10^9^	2.0 × 10^9^	1.1	N/A	N/A
NO	10	200	7.8	2.6 × 10^8^	1.3 × 10^9^	0.2	N/A	N/A
*Cs* H-NOX								
O_2_	7	200	63.7	2.6 × 10^8^	1.1 × 10^8^	2.4	4.3 × 10^7^	1.9
NO	1	200	1.42	3.8 × 10^7^	7.1 × 10^8^	0.053	1.5 × 10^8^	3.4 × 10^−3^
CO	-	-		-	-	-	3.3 × 10^6^	0.5
myoglobin (calculated data from ref. [22])								
O_2_ [34]	N/A	N/A		N/A	N/A		1.7 × 10^7^	15
NO [35]	15	300		1.8 × 10^8^	8 × 10^7^		1.7 × 10^7^	1.2 × 10^−4^
CO [34]	21	635		1.1 × 10^8^	2.5 × 10^8^		5.1 × 10^5^	1.9 × 10^−2^

**Table 2:** Global descriptors of gas molecules–protein interactions derived from the MD trajectories for bacterial H-NOX systems.

System	NO	O_2_	CO
**Average Number of Gas Molecules in the Protein**			
*Cs*	9.4	9.5	7.0
*Ka*	16.4	9.8	7.6
*Ns*	17.6	11.4	8.2
**Average Number of Protein Contacts Summed over All Gas Molecules in the Protein Matrix**			
*Cs*	70.2	103.0	44.0
*Ka*	104.0	95.6	45.0
*Ns*	117.2	99.1	47.9
**Average Number of Protein Contact/Gas Molecule**			
*Cs*	7.5	10.8	6.3
*Ka*	6.3	9.7	6.0
*Ns*	6.7	8.7	5.8

**Table 3:** Identified binding cavities in *Ns/Ka/Cs* H-NOX systems. Occupancy by at least one ligand molecule over the trajectory is given in %. In the case of the NO-containing systems, the average number and the observed maximum number of NO molecules in the pockets are also shown. For the distal pockets further information is given. The spatial distribution of the pockets is depicted in Figure 5.

Binding Pockets	NO	O_2_	CO
***Cs H-NOX***			
Distal pocket:	99.3%/1.32/4	92.8%	91.13%
average distance of centroid of gas molecule—FE	4.0 Å	3.95 Å	4.3 Å
average length of being trapped	920.2 ps	189.8 ps	609.0 ps
number of in-and-out event	323	1469	463
Proximal pocket:	47.90%/1.09/2	28.6%	0.0%
**Tunnel 1** (Xe2 and Xe3):			
Xe2:	89.6%/1.08/4	50.7%	65.2%
Xe3:	92.4%/1.21/4	57.5%	56.1%
**Tunnel 2** (Side-propionate):	96.0%/1.51/4	40.4%	24.0%
**Tunnel 3** (Side-entrance):	27.8%/1.08/3	18.0%	16.6%
Surface pockets (SP):			
Distal surface pocket (DSP):	75.8%/1.00/1	38.3%	34.2%
Proximal surface pocket 1 (P1SP) (αF, αE):	66.8%/1.02/2	55.7%	45.4%
Proximal surface pocket 2 (P2SP) (αF, β-sheet):	99.25%/1.00/2	9.0%	12.8%
***Ka H-NOX***			
Distal pocket:	86.1%/1.32/4	42.2%	13.5%
average distance of centroid of gas molecule—FE	5.3 Å	5.1 Å	5.6 Å
average length of being trapped	116.0 ps	59.0 ps	45.0 ps
number of transfer events in-and-out	2258	2255	922
Proximal:	99.95%/1.00/2	75.1%	99.44%
**Tunnel 1** (Xe2 and Xe3):			
XE2:	87.64%/1.10/4	26.4%	55.65%
XE3:	95.6%/1.11/3	37.9%	76.3%
**Tunnel 2** (Side-propionate):	29.2%/1.04/3	10.7%	16.67%
**Tunnel 3** (Side-entrance):	86.6%/1.20/4	18.2%	22.0%
Buried pockets/cavities (BP):BP1:	94.8%/1.21/3	21.3%	13.4%
***Cs H-NOX***			
Distal pocket:	64.8%/1.2/2	44.4%	0.0%
average distance of centroid of gas molecule—FE	4.3 Å	3.6 Å	-
average length of being trapped in distal pocket	27.5 ps	86.6 ps	-
Proximal pocket:	3.3%/1.00/1	56.9%	0.4%
Accessible-binding sites in the diffusion tunnels:	82.7%/1.00/2	22.2%	75.1%
**Tunnel 2** (Side-propionate):			
Proximal surface pocket 1 (P1SP) (αF, αE):	10.2%/1.01/2	12.8%	84.4%
Buried pockets/cavities (BP):			
BP1:	0.2%/1.00/1	69.5%	0.0%
BP2:	5.8%/1.00/2	28.5%	99.3%
BP3:	39.5%/1.00/1	4.0%	0.0%

## Supplementary Materials

Supplementary Materials are available online.

## Abbreviations

H-NOX	Heme-Nitric oxide and OXygen binding domain
*Cs or Tt*	*Caldanaerobacter subterraneus or Thermoanaerobacter tengcongensis*
*Ns*	*Nostoc* sp.
*Ka*	*Kordia algicida*
MD	molecular dynamics
sGC	soluble guanylate cyclase
NO	nitric oxide
CO	carbon monoxide
XO	CO or NO or O_2_
RMSF	root mean square fluctuation
RMSD	root mean square deviation
P1SP	Proximal surface pocket 1
P2SP	Proximal surface pocket 2
BP1	Buried pocket 1
BP2	Buried pocket 2
BP3	Buried pocket 3
Xe2	Xe-binding pocket 2
Xe3	Xe-binding pocket 3
DSP	Distal surface pocket
SP	Surface pocket
OPC	Optimal point charge water model
