# Supplementary material for: Gas Sensing by Bacterial H-NOX Proteins: An MD Study
Source: Molecules. 2020 Jun 23;25(12):2882. doi: 10.3390/molecules25122882 (PMC7356049; doi:10.3390/molecules25122882)
Supplement: Supplementary file 1 [file molecules-25-02882-s001.pdf]

Supplementary Materials for

Gas-sensing by bacterial H-NOX proteins: an MD study

Ahmed M. Rozza,[1,2] Dóra K. Menyhárd[3,*] and Julianna Oláh[1,*]

[1] Department of Inorganic and Analytical Chemistry, Budapest University of Technology, Budapest Szent Gellért tér 4. H-1111 ahmed.rozza89@azhar.edu.eg
[2] Department of Biotechnology, Faculty of Agriculture, Al-Azhar University, Cairo 11651, Egypt
[3] Laboratory of Structural Chemistry and Biology & MTA-ELTE Protein Modelling Research Group, Institute of Chemistry, Eötvös Loránd University, Pázmány Péter sétány 1/A, 1117 Budapest, Hungary
* Correspondence: dmenyhard@chem.elte.hu and olahjucsi@gmail.com

I. Ligand parameters used in the molecular dynamics simulations
II. **Table S1**. Overall RMSD values of backbone atoms calculated for the various trajectories
III. **Table S2**. Identified binding cavities (with residue numbers) and selected properties (overall occupancy in % of the last 300 ns of the whole trajectory, average distance of the centroid of the gas molecule from the heme iron, average length of a molecule being trapped, number of in-and-out events) in the case of NO, O_2 and CO diffusion to *Ns* H-NOX. The most fluctuating amino acid residues of each pocket are highlighted in bold.
IV. **Table S3**. Identified binding cavities (with residue numbers) and selected properties (overall occupancy in % of the last 300 ns of the whole trajectory, average distance of the centroid of the gas molecule from the heme iron, average length of a molecule being trapped, number of in-and-out events) in the case of NO, O_2 and CO diffusion to *Ka* H-NOX. The most fluctuating amino acid residues of each pocket are highlighted in bold.
V. **Table S4**. Identified binding cavities (with residue numbers) and selected properties (overall occupancy in % of the last 300 ns of the whole trajectory, average distance of the centroid of the gas molecule from the heme iron, average length of a molecule being trapped, number of in-and-out events) in the case of NO, O_2 and CO diffusion to *Ns* H-NOX. The most fluctuating amino acid residues of each pocket are highlighted in bold.
VI. **Table S5**. Calculated gas and protein concentrations and cell size in the MD simulations
VII. **Figure S1.** Blocking of Tunnel 3 in *Ns* H-NOX by the hydrogen bond between TRP74 and heme group.
VIII. **Figure S2.** Blocking of Tunnel 1 in *Cs* H-NOX by the hydrogen bond triad among TYR140, TRP9 and ASN74; steric hindrance of the tunnel by the hydrophobic interaction between two bulky residues (TRP9 and TRP67).
IX. **Figure S3.** Blocking of Tunnel 2 in *Ka* H-NOX by (a) the cation-π interaction between PHE45 and ARG 137 and (b) the steric hindrance caused by MET1 and MET 139.

Ligand parameters used in the molecular dynamics simulations

NO-ligand [44]

Bonded parameters:

[atoms]

; id	at type	res nr	residu name	at name	charge
1	LP	1	NO	DU	0.595
2	NO	1	NO	N	-0.250
3	ON	1	NO	O	-0.345

[bonds]

; i	j	funct	length	force.c.
2	3	1	0.1151	960228

; Vsite from			funct	a
1	2	3	1	0.53333

Non-bonded Parameters:

at type	Sigma (nm)	epsilon (kJ/mol)
NO	0.32786885250	0.8368
ON	0.33606557380	0.66944
LP	0	0

CO-ligand [32]

Bonded parameters:

[atoms]

; id	at type	res nr	residu name	at name	cg nr	charge
1	LP	1	CO	DU	1	1.600
2	CM	1	CO	C	1	-0.750
3	OM	1	CO	O	1	-0.850

[bonds]

; i	j	funct	length	force.c.
2	3	1	0.1128	1115000

; Vsite from			funct	a
1	2	3	2	0.5000

Non-bonded Parameters:

at type	Sigma (nm)	epsilon (kJ/mol)
CM	0.383000000000	0.10963
OM	0.312000000000	0.66567
LP	0	0

O_2-ligand [30]

Bonded parameters:

```
[ atoms ]
; id    at.type  res.nr   residue   at.name  cg.nr      charge
1         LPX      1       OXY         LP        1        0.228
2         OX       1       OXY         OX1       1       -0.114
3         OX       1       OXY         OX2       1       -0.114

[ bonds ]
; i    j          funct       length        force.c.
  2    3            2          0.121         1.4559e+07

; Vsite from                  funct        a
1      2      3                  1          0.5000
```

Non-bonded Parameters:

**at type**	**Sigma (nm)**	**epsilon (kJ/mol)**
OX	0.205000000000	0.6656744 ;
LPX	0.300578000000	0.43046 ;

Table S1. Overall RMSD values of backbone atoms calculated for the various trajectories

	**NO**			**CO**			**O_2**			**Apo**		
**H-NOXs**	***Ns***	***Ka***	***Cs***	***Ns***	***Ka***	***Cs***	***Ns***	***Ka***	***Cs***	***Ns***	***Ka***	***Cs***
**Ave. RMSD [nm]**	0.116	0.155	0.279	0.109	0.137	0.132	0.128	0.146	0.297	0.143	0.164	0.240

Table S2. Identified binding cavities (with residue numbers) and selected properties (overall occupancy in % of the last 300 ns of the whole trajectory, average distance of the centroid of the gas molecule from the heme iron, average length of a molecule being trapped, number of in-and-out events) in the case of NO, O_2 and CO diffusion to *Ns* H-NOX. The most fluctuating amino acid residues of each pocket are highlighted in bold.

**Binding Pockets**	**NO**	**O_2**	**CO**
➢ **Distal pocket:** [**<u>MET1</u>**, VAL5, TRP74, **<u>MET144</u>**, heme]	99.3%	92.8%	91.13%
- average distance of centroid of gas molecule---FE	4.0 Å	3.95 Å	4.3 Å
- average length of being trapped	920.2ps	189.8ps	609.0ps
- number of *in-and-out* event	323	1469	463
➢ **Proximal pocket:** [**<u>LEU104</u>**, HIS105, **<u>VAL108</u>**, GLY109, LEU115, PRO118, heme]	47.90%	28.6%	0.0%
- average distance of centroid of gas molecule---NE2 of HIS105	4.94 Å	4.5Å	-
- average length of being trapped	212.2ps	40.1ps	-
- number of *in-and-out* event	677	2144	-
➢ **Accessible-binding sites in the diffusion tunnels:**			
**Tunnel 1 (Xe2 & Xe3):**			
***Xe2:** [VAL5, **<u>ILE9</u>**, THR48, LEU67, PHE70, **<u>LEU141</u>**]	89.6%	50.7%	65.2%
- average length of being trapped	62.0ps	21.2ps	63.8ps
- number of transfer events *in-and-out*	4335	7155	3162
***Xe3:** [ILE9, MET12, ILE13, VAL52, **<u>LEU66</u>**, PHE70]	92.4%	57.5%	56.1%
- average length of being trapped	185.9ps	40.4ps	133.6ps
- number of transfer events *in-and-out*	1488	4284	1300
**Tunnel 2 (side-propionate):**			
[**MET1**, THR48, **TYR49**, LEU67, **ARG138**, CYS139, GLY140, **LEU141**]	96.0%	40.4%	24.0%
- average length of being trapped	144.0ps	21.4ps	16.9ps
- number of transfer events *in-and-out*	2001	5690	4423
**Tunnel 3 (Side-entrance):**			
[LEU4, TYR83, LEU86, SER111, PHE112]	27.8%	18.0%	16.6%
- average length of being trapped	17.2ps	19.3ps	17.6ps
- number of transfer events *in-and-out*	4857	2801	2928
➢ **Surface pockets (SP):**			
***Distal surface pocket (DSP)** :			
[GLN10, TRP22, LYS26, LEU31, **<u>ILE34</u>**, **<u>ASP35</u>**, **<u>PHE36</u>**, PHE37]	75.8%	38.3%	34.2%
- average length of being trapped	3913ps	304.0ps	1858.3ps
- number of transfer events *in-and-out*	57	378	57
***Proximal surface pocket 1** (P1SP) **(αF, αE):**			
[**<u>LEU86</u>**, SER89, ALA90, ASN100, HIS104, **<u>ARG107</u>**]	66.8%	55.7%	45.4%
- average length of being trapped	139.5ps	131.0ps	102.45ps
- number of transfer events *in-and-out*	1437	1276	1370
***Proximal surface pocket 2 (P2SP) (α F, β-sheet):**			
[PRO95, MET98, **<u>GLU99</u>**, CYS122, **<u>HIS124</u>**, MET130]	99.25%	9.0%	12.8%
- average length of being trapped	882.7ps	86.1ps	302.4ps
- number of transfer events *in-and-out*	337	314	130

Table S3. Identified binding cavities (with residue numbers) and selected properties (overall occupancy in % of the last 300 ns of the whole trajectory, average distance of the centroid of the gas molecule from the heme iron, average length of a molecule being trapped, number of in-and-out events) in the case of NO, O_2 and CO diffusion to *Ka* H-NOX. The most fluctuating amino acid residues of each pocket are highlighted in bold.

Binding Pockets	NO	O_2	CO
➢ **Distal pocket:** [**MET1**, **ILE5**, PHE74, PHE142, MET139, heme]	86.1%	42.2%	13.5%
- average distance of centroid of gas molecule---FE	5.3 Å	5.1 Å	5.6 Å
- average length of being trapped	116.0ps	59.0ps	45.0ps
- number of transfer events *in-and-out*	2258	2255	922
➢ **Proximal:** [HIS104, GLU106, VAL107, ARG108, ALA114, **GLU115**, LEU116, heme]	99.95%	75.1%	99.44%
- average distance of centroid of gas molecule---NE2 of HIS104	5.3 Å	5.3 Å	5.3 Å
- average length of being trapped	7940 ps	284.5 ps	2277ps
- number of transfer events *in-and-out*	37	791	118
➢ **Accessible-binding sites in the diffusion tunnels:**			
**Tunnel 1 (Xe2 & Xe3):**			
***XE2:** [ILE5, PHE9, MET48, LEU52, LEU67, **TYR70**, MET139]	87.64%	26.4%	55.65%
- average length of being trapped	84.3ps	19.2ps	75.0ps
- number of transfer events *In-and-out*	3165	4363	2282
***XE3**: [PHE9, LEU12, VAL13, PHE17, **LEU66**, THR69, **TYR70**]	95.6 %	37.9%	76.3%
- average length of being trapped	214.2ps	50.0ps	332.5ps
- number of transfer events *in-and-out*	1337	2275	693
**Tunnel 2 (Side-propionate):**			
[MET1, PHE45, MET48, LEU49, LEU67, ARG137, MET139]	29.2%	10.7%	16.67%
- average length of being trapped	13.0ps	8.0ps	9.5ps
- number of transfer events *in-and-out*	6844	4237	5387
**Tunnel 3 (Side-entrance):**			
[**ILE4**. VAL77, ILE78, SER81, **TYR82**, VAL107, TYR111, heme part (CMA, CBB and CMB)]	86.6%	18.2%	22.0%
- average length of being trapped			55ps
- number of transfer events *in-and-out*	73ps	34ps	1178
	3542	1571	
➢ **Buried pockets/cavities (BP):**			
**BP1:** [LEU96, PHE119, **MET131**, TYR133, HIS140, GLY143, LEU144, MET147, **PHE174**]	94.8%	21.3%	13.4%
- average length of being trapped	184.0ps	17.3ps	69.0ps
- number of transfer events *in-and-out*	1555	3902	597

Formatted Table

Table S4. Identified binding cavities (with residue numbers) and selected properties (overall occupancy in % of the last 300 ns of the whole trajectory, average distance of the centroid of the gas molecule from the heme iron, average length of a molecule being trapped, number of in-and-out events) in the case of NO, O_2 and CO diffusion to *Cs* H-NOX. The most fluctuating amino acid residues of each pocket are highlighted in bold.

Binding Pockets	NO	O_2	CO
➢ **Distal pocket:** [**MET1**, **ILE5**, PHE78, TYR140, heme]	64.8%	44.4%	0.0%
- average distance of centroid of gas molecule---FE	4.3 Å	3.6 Å	-
- average length of being trapped in distal pocket	27.5ps	86.6ps	-
➢ **Proximal pocket:** [**PHE94**, **MET98**, VAL101, HIS102, LEU105, THR106, PRO115, LEU117, heme]	3.3%	56.9%	0.4 %
-average distance of centroid of gas molecule---NE2 of HIS102	5.1Å	4.7Å	??
➢ **Accessible-binding sites in the diffusion tunnels:** **Tunnel 2 (Side-propionate):** [**MET1**, TRP9, **ASP45**, VAL48, **ARG49**, TRP67, ARG135, MET137]	82.7%	22.2%	75.1%
➢ **Surface pockets (SP):** ***Proximal surface pocket 1 (P1SP) (α F, β-sheet):** [**PHE86**, ARG89, PHE94, MET97, **MET98**, VAL101]	10.2%	12.8%	84.4%
➢ **Buried pockets/cavities (BP):** **BP1:** [**MET98**, LEU117, **MET129**, GLU130, TYR131, PHE141, LEU142, ILE145, LEU172, VAL174]	0.2%	69.5%	0.0%
**BP2:** [LEU95, **MET98**, **MET129**, PHE141, LEU144, ILE145]	5.8%	28.5%	99.3%
**BP3:** [VAL6, TRP9, ILE10, LEU13, VAL48, ILE51, **PHE52**, TRP67]	39.5%	4.0%	0.0%

Table S5. Calculated gas and protein concentrations and cell size in the MD simulations

H-NOX	[Gas]	[Protein]	Unit cell volume
Ns	0.176 mol/dm^3	0.00440 mol/dm^3	3.77545E-22 dm^3
Ka	0.173 mol/dm^3	0.00431 mol/dm^3	3.84863E-22 dm^3
Cs	0.133 mol/dm^3	0.00332 mol/dm^3	5.00349E-22 dm^3

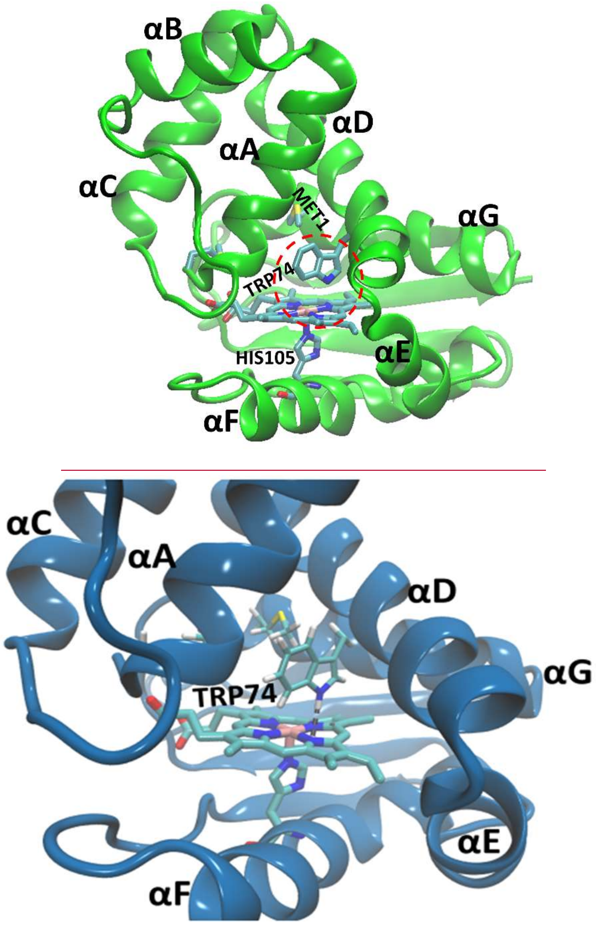

Figure S1. Blocking of Tunnel 3 in *Ns* H-NOX by the hydrogen bond between TRP74 and heme group.

Formatted: Font: Italic

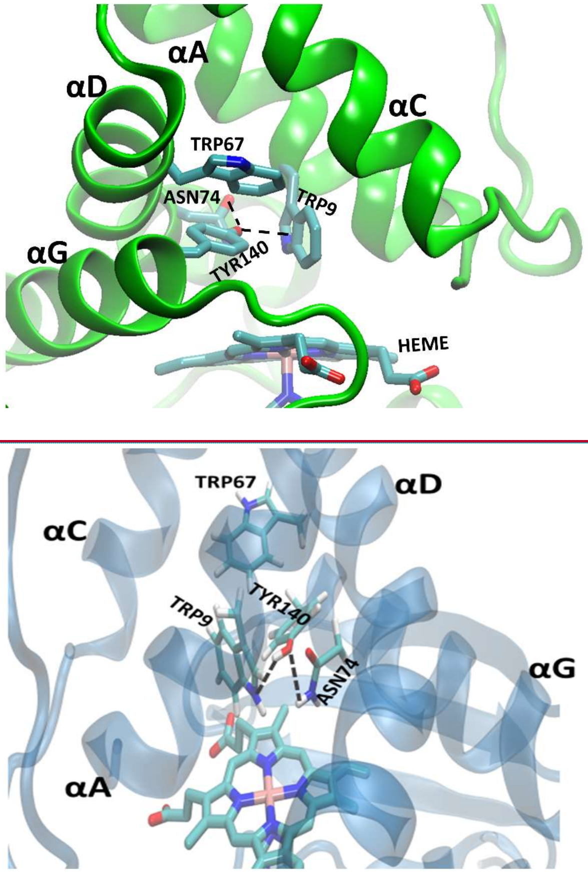

Figure S2. Blocking of Tunnel 1 in Cs H-NOX by the hydrogen bond triad among TYR140, TRP9 and ASN74; steric hindrance of the tunnel by the hydrophobic interaction between two bulky residues (TRP9 and TRP67).

Formatted: Font: Bold

(a) (b)

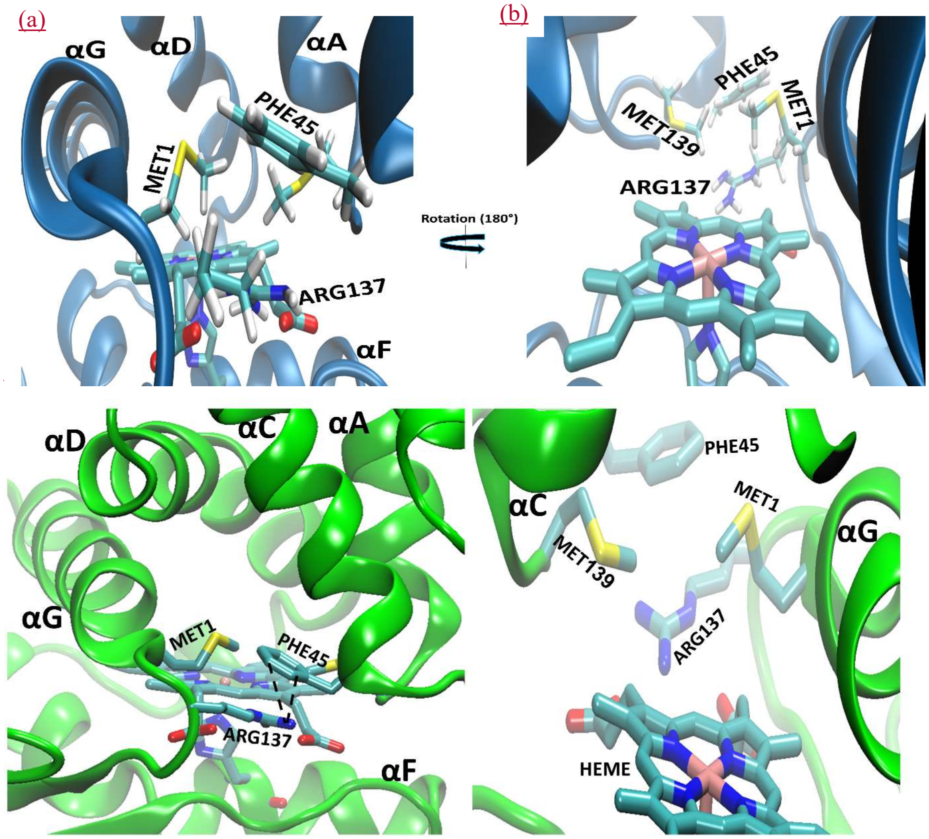

Figure S3. Blocking of Tunnel 2 in *Ka* H-NOX by (a) the cation-π interaction between PHE45 and ARG 137 and (b) the steric hindrance caused by MET1 and MET-139.

Formatted: Font: (Default) Palatino Linotype, (Asian) Times New Roman, 10 pt, Bold, Font color: Black
